# Early Obesity Prevention: A Randomized Trial of a Practice-Based Intervention in 0–24-Month Infants

**DOI:** 10.1155/2015/795859

**Published:** 2015-05-11

**Authors:** Natalia Schroeder, Berenice Rushovich, Edward Bartlett, Sangita Sharma, Joel Gittelsohn, Benjamin Caballero

**Affiliations:** ^1^Center for Human Nutrition, Bloomberg School of Public Health, Johns Hopkins University, Baltimore, MD 21205, USA; ^2^University of Maryland, School of Social Work, Ruth H. Young Center for Families and Children, Baltimore, MD 21201, USA; ^3^Johns Hopkins Community Physicians, Johns Hopkins Medicine, Baltimore, MD 21211, USA; ^4^Aboriginal and Global Health Research Group, Department of Medicine, Faculty of Medicine & Dentistry, University of Alberta, Edmonton, AB, Canada

## Abstract

*Objective*. A pediatric office-based intervention was implemented following a randomized, controlled design, aimed at improving child feeding practices and growth patterns and ultimately reducing risk for overweight and obesity later in life. *Methods*. Four clinics (232 infants) were randomized to control or intervention (I), the latter delivered by health care provider at each of 7–9 well-baby visits over 2 years, using a previously developed program (Growing Leaps and Bounds) that included verbal, visual, and text advice and information for parents. *Results*. The I group offered significantly less soda (*p* = 0.006), sweetened tea (*p* = 0.01), punch (*p* = 0.02) and/or cow's milk (*p* = 0.001) to infants and delayed the introduction of drink/food other than breast milk (*p* < 0.05). Parents in the I group had a higher perceived parental monitoring (*p* = 0.05) and restriction (*p* = 0.01) on infant feeding. While the I group exhibited at baseline more adverse socioeconomic indicators than the control group, growth trajectory or body size indices did not significantly differ between groups. *Conclusions*. Education provided by health care providers in addition to follow-up monthly phone calls may help modify parental behaviors related to child feeding and increase parental sense of responsibility toward child eating behaviors.

## 1. Introduction

Childhood obesity remains a major public health concern [1]. Approximately 17% of children and adolescents aged 2–19 years are obese [1], and 9.7% of infants and toddlers (birth to 2 years) have high weight-for-recumbent length (95th percentile or higher) [2]. Excess weight during childhood may increase risk of noncommunicable diseases in adulthood [3].

Obesity prevention trials have largely focused on schoolchildren or adolescents. However, data from observational studies suggest that rapid growth in the first 2 years of age may be associated with higher risk of obesity later in life [4–6]. Therefore, this period needs to be studied to explore opportunities for early prevention interventions.

To the best of our knowledge, there are no previously randomized controlled educational interventions starting at birth and centered on the health care provider. The present study was designed to assess whether an early, office-based, scalable intervention could affect the usual pattern of feeding and growth trajectory over the first 2 years of life.

## 2. Methods

The study was a randomized, cluster-stratified clinical trial carried out in 4 health centers from the Johns Hopkins Community Physicians (JHCP) network in Maryland. Two centers were included in the intervention group and the other two in the control group. Centers were balanced to include one urban and one suburban center in each group. The study was completed when all participants reached 24 months of age.

Inclusion criteria included all healthy newborns with ≥2000 g body weight and who were not requiring specialized medical or nutritional care and discharged home within 5 days after birth. The intervention was based on the modules of Growing Leaps and Bounds (GLB), a set of educational materials developed by a group of experts and funded by the Dannon Institute. These materials aim at (a) promoting an exchange between patient and pediatrician about nutrition, feeding, and physical activity; (b) providing useful information to parents in order to enhance self-efficacy for the daily care of their infants; and (c) helping parents make healthy food choices for the infants and for themselves and make physical activity a part of daily life.

The 12 sets of educational brochures were designed to be presented and discussed with caregivers at pediatric visits at 1, 2, 4, 6, 9, 12, 15, 18, and 24 months of age and at annual visits thereafter up to age 5 years. While the brochures emphasize a few key points, they also provide detailed advice on infant feeding practices, physical activity, and developmental milestones related to eating patterns.

All participating pediatricians, nurse practitioners, and clinic staff attended training sessions before start of the study. Refresher sessions were held every 2-3 months. In the intervention clinics, the sessions focused on intervention content and delivery and in control clinics on the logistics and data collection. Participating pediatricians signed a memorandum of agreement and received a compensation of $150 per infant enrolled.

All parents completed a brief exit interview after they saw the physician, as part of process evaluation data collection. In between visits, parents received a phone call every month, providing encouragement and answering questions. They also received reminder post cards which also contained short educational messages.

### 2.1. Anthropometry

Weight and height were measured in duplicate using Seca self-calibrating scales and Shorr stadiometers. Triceps and subscapular skinfolds were measured following the World Health Organization (WHO) protocol, using Lange calipers.

The scales used were self-calibrating and did not need additional calibration. All staff were trained on how to complete the various measurements and followed up with a gold standard check where one staff member completed a remeasure of the infant to check for agreement. This was completed approximately once a quarter. Two repeat measures were completed if the initial two measurements were more than a set amount apart.

### 2.2. Child Feeding Practices

We measured child feeding practices at 9 months, using an instrument developed for the Tips on Parenting Study (TOPS) (M. Black et al.) [7]. Parental control and restrictiveness were assessed using the Child Feeding Questionnaire (CFQ), at 12 and 24 months. The CFQ is a 31-item scale that examines caregivers' perceptions of their children's risk for weight and eating problems and caregiver control over feeding.

### 2.3. Dietary Assessment

We developed a Food Frequency Questionnaire (FFQ) specifically for this study. For this we performed a preliminary 24-hour dietary recall in a comparable population in Baltimore [8]. The FFQ was pilot-tested to identify foods that were consumed but were not reported in recalls, using a convenience sample of 16 participants, recruited from three of the four JHCP health care centers. Portion size was assessed using food models and familiar household utensils. Ten categories of frequency were used, ranging from “never” to “6 or more times per day” and covering the previous 30 days. FFQs were obtained at 6, 12, and 24 months, by trained, certified field staff.

### 2.4. Process Evaluation

Health care providers and study staff were evaluated on their compliance with intervention protocol and delivery of key intervention messages, including items such as delivery of the brochure to caregiver, discussion of front-page points of brochure, and reminder of key guidance items.

### 2.5. Data Analysis

All data collected in the field was entered into electronic files and verified by reentering every 10th form by a staff other than the one collecting the data.

Data were analyzed using Stata statistics/data analysis software (version 11.2, College Station, Texas, 2009). Student's *t*-test was used to compare paired groups (intervention versus control, urban versus suburban). Analysis of variance (ANOVA) was used to determine differences among the four sites. Median height, weight, and BMI were used for trajectory plots. Linear regression models were used to determine the potential influence of SNAP participation on outcome variables. Covariates of interest included SNAP participation, WIC participation, breastfeeding, race, and gender. Significance was defined at the *p* < 0.05 level.

## 3. Results

A total of 292 infants were enrolled and 232 completed the study. This was consistent with our predicted attrition rate of 20%. All clinics but one had retention rates above 80%. The clinic with low retention (67%) near Washington, DC, serves many military families, which tend to be relocated frequently.

The breakdown of number of infants for each health center (included in the final analysis) was as follows: Center 1: 63 (31 M, 32 F); Center 2: 49 (18 M, 31 F); Center 3: 57 (31 M, 26 F); and Center 4: 53 (28 M, 25 F). Ethnicity breakdown for the JHCP health care centers (2005-2006) was as follows: Black 48%, White 35%, Asian 2%, Hispanic 2%, Indian 0%, Multiracial 0%, others 6%, and unknown 7%.

The intervention group had higher number of African-American caregivers, higher unemployment rate, lower household income, lower completed education level, and less home ownership than the control group. The intervention group also used more food stamps and more WIC program services and had lower rates of breastfeeding. These characteristics were particularly driven by the urban intervention clinic.

### 3.1. Implementation

Process evaluation was conducted to assess the quality of implementation of the intervention. Two primary measurement tools were developed, one to assess quality of implementation by pediatricians and the other by our study staff. Physicians and study staff received a score for each intervention visit based on whether they provided the appropriate brochure to the participant and how well they reinforced the key messages of the brochure. Physicians exhibited consistently lower implementation scores than study interventionists, and these scores decreased over time, from an average of about 70% to less than 50%.

### 3.2. Impact on Anthropometry

Anthropometric data at baseline, 12 months, and final visit (24 months) is presented in Table 1. At 24 months of age, there was no effect of the intervention on height, weight, BMI, BMI *z*-scores, triceps skinfolds, or triceps + subscapular skinfolds. At baseline (1.4–2.0 months of age) weight and height were significantly higher in the intervention versus the control group (*p* < 0.006 and *p* < 0.002, resp.), but this difference disappeared at 12 and 24 months (*p* > 0.05). Triceps skinfold was higher in suburban clinics at 12 and 24 months but did not differ between intervention and control. A similar pattern was observed for the sum of triceps + subscapular skinfolds, although these results were only statistically significant at 12 months. All anthropometric results were similar when low birth weight (<2500 g) babies were excluded from the analyses.

Infant birth weight did not significantly differ between intervention and control (3251.7 g (565.02), 3220.8 g (480.72), resp.). However, infant birth weight significantly differed by clinic (EB 3191.8 g (460.13), OD 3318.0 g (660.58), WM 3358.7 g (483.72), and WP 3047.0 g (411.77)) and if the location represented urban or suburban (3123.4 g (443.64), 3341.1 g (567.00), resp.) (*p* < 0.0001). Additionally, white babies (3338 g) were significantly heavier than African-American babies (3107 g) at birth (*p* < 0.004).

**Figure 1:**
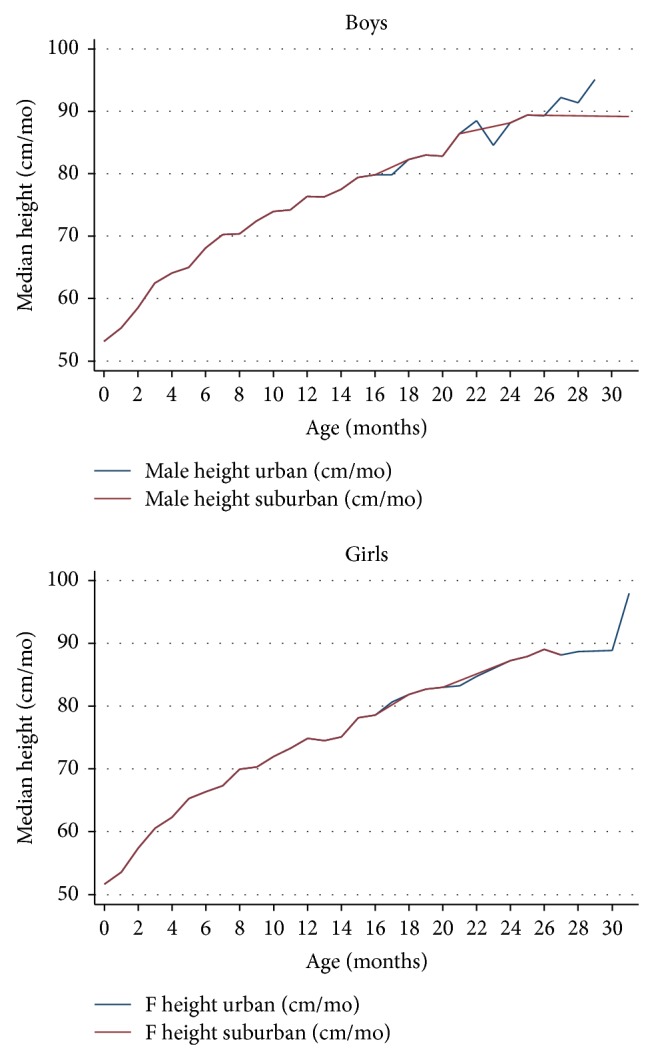
Growth trajectory (height) by geographic location for boys and girls.

Growth trajectories for both weight and height were similar in intervention-control and urban-suburban clinics and closely tracked the medians of the 2006 WHO reference charts (see Figure 1).

Obesity prevalence in our sample was low (0.6% to 0.4% for obesity and 3.8 to 4.3% for overweight at 6 and 24 months of age, resp.). Based on the Centers for Disease Control and Prevention (CDC) Growth Charts, if an infant was >85% BMI percentile, the infant was considered overweight and if an infant was >95% BMI percentile, then the infant was considered obese. The intervention group had a prevalence of overweight of 2.7 and 5.3% at 6 and 24 months, respectively. The control group had overweight prevalences of 4.7 and 3.2% at similar age periods. There was only one child classified as obese at the end of the study, in the intervention group.

### 3.3. Impact on Dietary Intake

**Figure 2:**
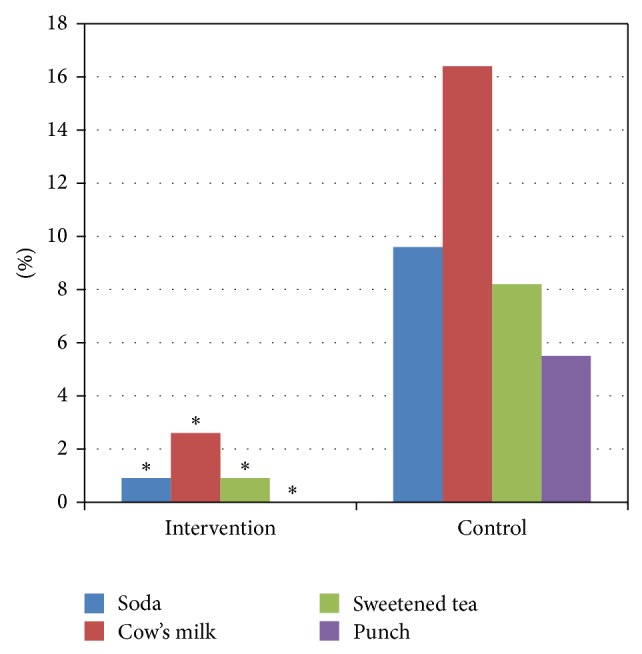
Liquids other than breast milk or formula offered to infants. ^∗^Soda: *p* < 0.006, cow's milk: *p* < 0.001, sweetened tea: *p* < 0.014, and punch: *p* < 0.021.

**Figure 3:**
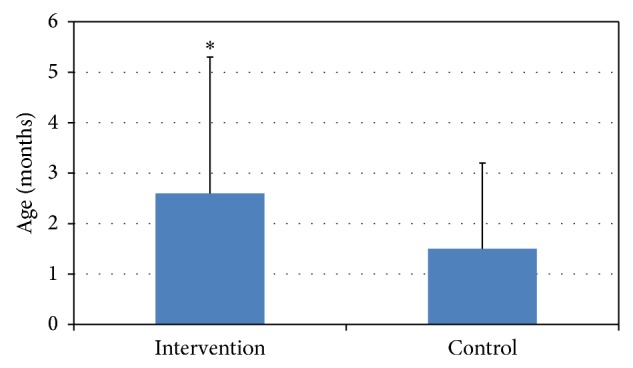
Age of infant at introduction of food/drink other than breast milk. ^∗^ *p* < 0.051. *n* = 82 (control = 37, intervention = 45), considering only caregivers who ever breastfed.

The intervention group was less likely to use infant cereal (*p* < 0.001) or stage 1 vegetables (*p* < 0.05) as the first complementary food. Also, the intervention group offered significantly less soda (*p* < 0.006), sweetened tea (*p* < 0.01), punch (*p* < 0.02), or cow's milk (*p* < 0.001) than the control group (Figure 2). The intervention group also delayed introduction of drink/food other than breast milk, compared with the control group (*p* < 0.05) (Figure 3). A comparison between 6 and 24 months indicated that the control group increased consumption of unsweetened drinks (*p* < 0.04) and of vitamin supplements (*p* < 0.04) relative to the intervention group.

### 3.4. Child Feeding Practices

The results of the CFQ are shown in Table 2. Parents in the intervention group exerted more dietary restriction on their child (*p* < 0.01) and were more active in monitoring child feeding (*p* < 0.05) than those in the control group. Overall, breastfeeding practice was low. Current breastfeeding rates as reported by mothers in the intervention and control group, respectively, were as follows: 21% and 18% at baseline, 23% and 20% at 6 months, 22% and 22% at 9 months, 21% and 19% at 12 months, and 20% and 20% at 24 months (final visit). In the intervention group, 49% of mothers reported ever breastfeeding versus 64% in the control group. The lower rate of ever breastfeeding reported was primarily driven by the urban intervention site.

### 3.5. Income Level and Supplemental Nutrition Assistance Program (SNAP)

SNAP participation was associated with income level and was substantially higher in urban (65 and 43%) than in suburban (6% in both) clinics, regardless of treatment allocation. Income level was similar in 3 of the 4 clinics: suburban C and suburban I and urban C. The urban I clinic had 74% of participants in the lowest income level, compared with 20–30% in the other three. We evaluated the possible association between SNAP use and study outcomes. The only significant difference was for body weight at 24 months, which was 0.7 kg higher in SNAP participants (*p* < 0.01).

## 4. Discussion

Our results show that a simple guidance program introduced during routine well-baby visits can positively change several parental dietary practices. For example, parents receiving the intervention provided less soda, sweetened tea, punch, or cow's milk as complementary fluids for breast-fed babies. Juice and sweetened beverage consumption may be associated with obesity and short stature further displacing nutrient-dense foods [9, 10]. Intervention parents also delayed the introduction of fluids or foods other than breast milk. Early introduction of foods continues to be a concern [11] and may increase the risk of obesity [12, 13].

Breastfeeding in our study population was lower than the state of MD average at 6 months (21% versus 48%), but comparable at 12 months (20% versus 22%). For those who ever breastfed, the rate for both the intervention (49%) and control (64%) was lower than the rate reported for the state of Maryland (72.6%). This difference may be due to our small sample size, clearly not representative of the diversity of the state of MD.

There were no differences in BMI between groups at the end of the intervention. However, the intervention group had significantly higher weight and height than the control group at the beginning of the study, and this difference disappeared at around 6 months. We did not adjust for baseline weight and height in the analysis. We used the Student's *t*-test to look at differences between intervention and control for the anthropometric measurements. Because of the sample size, it was important not to make too many adjustments.

Pediatricians and their health care team can greatly influence parental child rearing behaviors, but time constraints may limit opportunities for delivery and tracking of educational messages. The GLB program was designed to be presented in about 5 minutes, focusing on no more than three items at each visit and including a printed brochure as a permanent record of each mini session. As expected implementation was strong during the first year, when well-baby visits are frequent, and declined in the second year, when many children have only 2 or 3 visits.

We found only three studies on obesity prevention starting at birth [14–16] and a methods paper “as discussed by Karanja et al. [17].” Costom and Shore [14] focused on an individualized feeding approach, including individualized feeding instructions (exclusive breastfeeding and introduction of cow's milk and solid foods) based on the growth pattern of the infant starting at 6 weeks and at 3, 5, 8, 12, 16, and 20 months. Additionally, growth curves of the infant's height and weight were provided to parents to help them visualize their infant's growth; further, misconceptions about a child's weight gain were addressed when necessary. Costom and Shore's approach resulted in reduced adiposity in 182 infants and delayed introduction of foods until 3-4 months of age. In the current study, an approach customized to the infant's development significantly delayed the introduction of food and drink similar to Costom and Shore [14].

Karanja et al. [15] found the combination of a community-wide and family intervention in American Indian/Alaskan tribes attenuated BMI rise in toddlers (24 months old) and increased parents' confidence in reducing sugar-sweetened beverages. Similar to Karanja et al. [15], our data showed the intervention group exhibited greater dietary restriction and offered significantly less sweetened beverages to infants compared to the control group. However, this did not translate to changes in BMI. We may not have seen changes in BMI because our study population had a normal BMI. Karanja et al. found an attenuated rise in BMI in a population (American Indian) of infants that tend to have rapid growth from 6 to 9 months in age compared to WHO standards. On the other hand, Paul et al. [16] found infants (with a normal weight-for-length) had a lower weight-for-length percentile at 1 year after parents received two home visits focused on non-hunger-related fussiness, sleep duration, introduction of solid foods, and incorporation of healthy foods. According to the Pediatric Nutrition Surveillance System, 8.0% of 0–11-month-old infants and 12.3% of 24–35-month-old infants are considered obese [18]. The low prevalence of obesity in our sample may be due to the small sample size, further contributing to no effect of BMI.

One of our four study sites in the intervention group exhibited some of the demographic characteristics associated with low-income, inner city populations: higher unemployment, lower income, and more use of SNAP and WIC services. Because the pattern for income level mimicked the pattern for SNAP participation, we were interested in whether income level and SNAP participation influenced the various outcomes related to adiposity. Interestingly, babies whose families participated in SNAP had a final body weight 0.7 kg greater than those babies whose families did not participate in SNAP, the urban intervention site largely driving this finding. However, there was no difference in babies' BMI between families participating in SNAP versus nonparticipants. A recent study found, among low-income adults, that SNAP participation has been found to be associated with greater adiposity, BMI, waist circumference, and other metabolic risk factors [19]. Additionally, the diet quality of adult SNAP participants was lower than income-eligible nonparticipants [20]. Gibson 2006 found long-term food stamp participation (currently SNAP) to be positively associated with overweight in girls aged 4.5–11.5 years and obesity in mothers [21]. However, in preschool age children, BMI percentile did not differ between SNAP and non-SNAP participants although the prevalence of obesity was increased in SNAP participants compared to eligible nonparticipants but again were not statistically different [22]. Although our finding is somewhat controversial one must recognize the complexity of this outcome. Those who lived in the urban intervention site did not have access to adequate grocery stores and healthy food options, mainly purchasing food items from corner convenience stores where options are limited; thus, multiple and complex reasons may be contributing to the greater final body weight in those who participate in SNAP. When designing future interventions, one must take into consideration SNAP participation, urban dwelling, and socioeconomic status and customize the program appropriately. Further, because the physicians can have a major impact on parents' behavior, it is imperative that the physicians receive the most appropriate training for their clientele. Finally, it would be important to collect information on maternal anthropometry, medical history of the child, and pregnancy smoking in future interventions.

## 5. Conclusion

In summary, our intervention was able to significantly improve several parental behaviors related to child feeding and increased parental sense of responsibility toward their child's eating behaviors. While the small number of clinics included in the study resulted in an intervention group with more adverse socioeconomic indicators than the control group, there were no differences in growth trajectory or in body size indices between the groups at the end of the follow-up period. Education and motivation provided by health care providers may be an important component of a multilevel approach to early obesity prevention.

## Figures and Tables

**Table 1:** Anthropometric measurements of babies at baseline, 12 months, and final visit^a^.

	Baseline		12 months		Final visit	
	Intervention *n* = 134	Control *n* = 144	Intervention *n* = 105	Control *n* = 113	Intervention *n* = 112	Control *n* = 110
	Mean (SD) 95% confidence interval		Mean (SD) 95% confidence interval		Mean (SD) 95% confidence interval	
BMI	15.29 (1.53)	15.03 (1.58)	17.23 (1.58)	17.29 (1.95)	16.34 (1.59)	16.20 (1.25)
	15.04–15.56	14.77–15.29	16.93–17.54	16.93–17.66	16.05–16.65	15.96–16.44

BMI *z*-scores	−0.283 (0.96)	−0.152 (1.01)	0.492 (1.08)	0.539 (1.36)	0.339 (1.13)	0.218 (0.95)
	−0.45–−0.12	−0.32–0.01	0.29–0.70	0.29–0.79	0.13–0.55	0.04–0.40

Weight (kg)	4.91 (1.23)^∗^	4.56 (0.89)	9.85 (1.11)	9.81 (1.29)	12.76 (1.63)	12.61 (1.47)
	4.71–5.13	4.41–4.71	9.64–10.07	9.58–10.06	12.46–13.07	12.34–12.89

Height (cm)	56.30 (4.52)^∗∗^	54.81 (3.10)	75.53 (2.49)	75.31 (3.06)	88.20 (3.21)	88.13 (3.15)
	55.53–57.07	54.31–55.32	75.06–76.02	74.74–75.88	87.61–88.81	87.54–88.73

Triceps skinfold	7.94 (2.28)	7.85 (2.58)	9.70 (2.09)^∗∗^	8.82 (1.93)	8.83 (2.02)	8.42 (2.19)
	7.56–8.34	7.43–8.28	9.30–10.12	8.44–9.20	8.44–9.23	7.97–8.87

Triceps + subscapular skinfold	14.36 (3.91)	14.45 (4.01)	16.46 (3.52)^∗∗∗^	15.36 (2.99)	14.68 (3.25)	14.06 (3.17)
	13.70–15.04	13.78–15.12	15.78–17.15	14.78–15.95	14.04–15.33	13.40–14.72

^a^Student's *t*-test was used to compare intervention versus control at baseline, 12 months, and final visit.

^∗^ *p* < 0.006, ^∗∗^ *p* < 0.002, and ^∗∗∗^ *p* < 0.018.

**Table 2:** Child Feeding Questionnaire Scores by control and intervention groups at 24 months.

	Control *n* = 102	Intervention *n* = 84	*p* value
	Mean (SD) 95% confidence interval	Mean (SD) 95% confidence interval	
(1) Perceived feeding responsibility	4.45 (0.78)	4.50 (0.63)	0.930
	4.30–4.60	4.37–4.63	

(2) Perceived parent overweight	3.28 (0.50)	3.15 (0.50)	0.409
	3.18–3.38	3.04–3.26	

(3) Perceived child overweight	2.89 (0.47)	2.98 (0.26)	0.194
	2.80–2.98	2.92–3.04	

(4) Concerns about child overweight	2.06 (1.20)	2.29 (1.36)	0.329
	1.83–2.29	2.00–2.58	

(5) Dietary restriction	*3.44* (*0.86*)	*3.77* (*0.72*)	*0.010 *
	*3.27–3.61 *	*3.62–3.92 *	

(6) Pressure to eat	2.68 (1.10)	2.72 (1.30)	0.939
	2.47–2.89	2.44–3.00	

(7) Monitoring	*4.13* (*0.99*)	*4.41* (*0.84*)	*0.046 *
	*3.94–4.32 *	*4.23–4.59 *	

## Abbreviations

ANOVA: Analysis of variance
BAZ: BMI-for-age *z*-score
BMI: Body mass index
CFQ: Child Feeding Questionnaire
F: Female
FFQ: Food Frequency Questionnaire
GLB: Growing Leaps and Bounds
I: Intervention group
JHCP: Johns Hopkins Community Physicians
M: Male
MD: State of Maryland
SNAP: Supplemental Nutrition Assistance Program
WHO: World Health Organization
WIC: Women, Infants, and Children
WHZ: Weight-for-length *z*-score.
